# The Role of Artificial Intelligence Large Language Models in Personalized Rehabilitation Programs for Knee Osteoarthritis: An Observational Study

**DOI:** 10.1007/s10916-025-02207-x

**Published:** 2025-06-03

**Authors:** Ömer Alperen Gürses, Anıl Özüdoğru, Figen Tuncay, Caner Kararti

**Affiliations:** 1 School of Physical Therapy and Rehabilitation, Department of Physiotherapy and Rehabilitation, Kırşehir Ahi Evran University, Merkez, Kırşehir, 40100 Türkiye; 2 Faculty of Medicine, Department of Physical Medicine and Rehabilitation, Kırşehir Ahi Evran University, Merkez, Kırşehir, 40100 Türkiye

**Keywords:** Artificial intelligence, Large language models, Physiotherapy, Rehabilitation program, Knee osteoarthritis

## Abstract

**Background:**

Large language models (LLMs) can contribute to treatment options and outcomes by assisting physiotherapists for conditions like osteoarthritis.

**Aims:**

The objective of this early-stage cross-sectional study is to assess the alignment of large language models with physiotherapists in designing physiotherapy and rehabilitation programs for knee osteoarthritis.

**Methods:**

Forty patients diagnosed with knee osteoarthritis were assessed using standardized clinical criteria. For each patient, individualized rehabilitation programs were created by three physiotherapists and by ChatGPT-4o and Gemini Advanced using structured prompts. The presence or absence of 50 clinically relevant rehabilitation parameters was recorded for each program. Chi-square tests were used to evaluate agreement rates between the LLMs and the physiotherapist-generated Consensus programs.

**Results:**

ChatGPT-4o achieved a 74% agreement rate with the physiotherapists’ Consensus programs, while Gemini Advanced achieved 70%. Although both models showed high compatibility with general rehabilitation components, they demonstrated notable limitations in exercise specificity, including frequency, sets, and progression criteria. ChatGPT-4o performed as well as or better than Gemini in most phases, particularly in Phase 3, while Gemini showed lower consistency in balance and stabilization parameters.

**Conclusions:**

ChatGPT-4o and Gemini Advanced demonstrate promising potential in generating personalized rehabilitation programs for knee osteoarthritis. While their outputs generally align with expert recommendations, notable gaps remain in clinical reasoning and the provision of detailed exercise parameters. These findings underscore the importance of ongoing model refinement and the necessity of expert supervision for safe and effective clinical integration.

**Supplementary Information:**

The online version contains supplementary material available at 10.1007/s10916-025-02207-x.

## Introduction

Large language models (LLMs), a branch of artificial intelligence (AI), are advanced systems that leverage deep learning algorithms to process natural language and generate responses with human-like quality and consistency [1]. Notable LLMs such as ChatGPT and Gemini represent leading models in this field [2]. ChatGPT, developed by OpenAI, is based on the GPT architecture (versions 3.5 or 4.0) and functions both as a chatbot and a generative model, trained on multilingual datasets [3]. Gemini, in contrast, offers real-time data integration and is designed to interact with search engines, potentially reshaping information-seeking behavior [2].

The advent of LLMs has precipitated a surge in research endeavors exploring their potential applications in healthcare, clinical practice, and medical research [4, 5]. While conventional AI has exhibited limited involvement in clinical decision-making, LLMs, trained on extensive and diverse human-generated datasets, have catalyzed growing interest in their role in supporting clinical workflows, encompassing triage, diagnosis, and treatment planning [6]. Recent studies have explored the application of LLMs in osteoarthritis management, where models like ChatGPT have demonstrated moderate success in generating rehabilitation programs and aligning with clinical guidelines [7, 8]. Similar research in stroke rehabilitation has shown that LLMs are capable of mimicking clinical reasoning and creating structured treatment plans based on established principles [9]. Furthermore, investigations into conditions such as vestibular disorders, scoliosis, and musculoskeletal diagnostics suggest that these models may aid clinicians in patient communication, education, and treatment planning, particularly when supported by appropriate professional oversight [10–15]. By presenting medical information in a clear and patient-specific manner, LLMs can support clinicians in effectively communicating physiotherapy plans and explaining underlying conditions. This personalised approach may enhance patient understanding, improve adherence to treatment recommendations, and contribute to better health outcomes [16].

Osteoarthritis (OA) is one of the most common musculoskeletal diseases worldwide and has a significant effect on quality of life [17]. OA is a major focus of rehabilitation and multidisciplinary treatment approaches. By supporting both patients and physiotherapists, LLMs can contribute to better understanding, improved treatment strategies, and optimized outcomes for conditions like OA, addressing the needs of a broad patient population [18]. In particular, their potential to delineate fundamental rehabilitation strategies and lucidly expound treatment alternatives could assist physiotherapists in diminishing the time expended on repetitive documentation and preliminary programme design. Moreover, they could serve as a cautionary mechanism for interventions that are frequently overlooked during the planning process. Although studies on LLMs are present, research remains limited in two key areas: first, there are few studies of recent versions that outperform previous versions [7, 18, 19], and second, there are few studies that focus specifically on rehabilitation [9, 16, 20, 21]. This study represents an early-stage evaluation of large language model-based decision support tools by examining their alignment with physiotherapists in the development of personalized rehabilitation programs for knee osteoarthritis, thereby addressing a current gap in the literature.

## Materials and Methods

### Study Design

This cross-sectional study compared the physiotherapy programs developed by three experienced physiotherapists with at least five years of clinical experience in knee OA and those generated by ChatGPT-4o and Gemini Advanced, two large language models, for knee OA. The study was conducted between August and October 2024 at the physiotherapy outpatient clinic. The study followed the Strengthening the Reporting of Observational Studies in Epidemiology (STROBE) and Reporting guideline for the early stage clinical evaluation of decision support systems driven by AI (DECIDE-AI) [22] to ensure high-quality reporting standards.

### Participants

The study included 40 patients diagnosed with knee OA based on the American College of Rheumatology criteria by a physiatrist. The patients were aged between 40 and 65 years and diagnosed with grade 2 or 3 knee OA based on the Kellgren-Lawrence classification [23]. Exclusion criteria were previous knee surgery or joint injections within the last 6 months, history of any physiotherapy program, cognitive impairment, systemic diseases, neurological or orthopedic conditions affecting the lower extremities [24].

### Measurements

Data on age, sex, body mass index (BMI), and educational status were recorded for all patients. Pain was evaluated using the numeric pain rating scale. The range of motion of the hip and knee joints in all directions was measured with a universal goniometer (Baseline-12-1000 Plastic 360 Degree ISOM), and the strength of the quadriceps and hamstring muscles was assessed using an isometric dynamometer (Lafayette Hand-Held Dynamometer). Functional status was assessed using the WOMAC and Lysholm scores. Physical performance was measured with the Timed Up and Go test, 40-meter fast walk test, 30-second sit-to-stand test, and stair climb test [25, 26]. Balance assessments included the single-leg stance test for static balance and the Four Square Step Test for dynamic balance. The selection of these performance tests aligns with recommendations from the Osteoarthritis Research Society International (OARSI), which endorses their use as standard reliable measures for evaluating functional outcomes in hip and knee OA [26].

### Procedure

Two distinct approaches were used to create patient-based assessment and rehabilitation programs for all participants: one based on physiotherapist consensus and the other generated by LLMs.

Consensus physiotherapy programs: The data for each patient was independently evaluated by three physiotherapists, who subsequently developed preliminary programs. Through structured discussion, the physiotherapists were able to reach a Consensus on the most appropriate treatment program for each patient, resulting in a single standardized program for each patient.

AI-generated physiotherapy programs: The data obtained from patients after the evaluation were entered into ChatGPT-4o and Gemini Advanced using prompts written in Turkish. The prompts were originally in Turkish to simulate real-world usage scenarios in Turkey, where the primary audience includes Turkish-speaking physiotherapists and patients. The English translation of the prompts is provided in the main text, while the original Turkish prompts and the prompts related to patient data, in both Turkish and English versions, are included in Supplementary Material 1 to ensure transparency.

The following English translation of the prompt was used:

*"*Prepare a detailed three-phase physiotherapy program for a knee OA patient based on the provided evaluation parameters. The program should include the following components*:

*Electrophysical agents: Specify appropriate modalities for each phase.*

*Thermal applications: Indicate whether hot or cold treatments are preferred based on the patient’s needs.*

*Exercise applications: Provide a detailed exercise program for each phase*,* including repetitions*,* sets*,* and positions.*

*Phase transition criteria: Define specific criteria for progressing between phases in terms of pain*,* edema*,* balance*,* range of motion (ROM)*,* muscle strength*,* and functionality.*

*Discharge criteria: Highlight the goals the patient should achieve by the end of the rehabilitation.*“*.

This structured prompt format was intentionally designed to guide the language models to generate outputs aligned with real-world physiotherapy program components. Therefore, the models were not producing responses entirely independently, but within a standardized and directive framework that ensured the inclusion of clinically relevant rehabilitation elements. ChatGPT-4o was prompted immediately after each patient’s evaluation. A new conversation was initiated for each case to ensure independence of responses. According to OpenAI, this is sufficient to prevent prior context from influencing the model’s output, even within the same session [1].

To ensure variety and avoid repetition, new conversations were initiated for each patient when querying ChatGPT-4 and Gemini Advanced. Each of the 40 patient profiles was individually presented to the language models, which were prompted to generate personalized rehabilitation programs tailored to the specific clinical characteristics of each case. For every rehabilitation parameter and phase, the presence or absence of a recommendation was recorded for each patient. These binary data were then aggregated to calculate the percentage of cases in which a given intervention was recommended, allowing for item-level comparison across models and with the Consensus group. Although reported as overall frequencies, the data structure was built on case-specific inputs and individualized AI responses. This approach ensured that each AI-generated rehabilitation plan was based on individualized clinical data, reflecting a tailored treatment structure for every patient.

Parameter selection: Initially, 58 parameters were identified across the rehabilitation programs developed by the physiotherapists and AI models. The final list of 50 parameters was determined based on their clinical relevance and frequency of application in knee OA rehabilitation, as supported by established guidelines and prior literature [27, 28]. Parameters that were deemed less relevant appeared only in one or two patient-specific outputs, or lacked support in evidence-based physiotherapy practices were excluded. This decision ensured that the analysis focused on the most meaningful and representative components of the rehabilitation programs, allowing for comparison grounded in widely accepted clinical standards. A detailed list of excluded parameters is provided in Supplementary Material 2.

### Sample Size

The sample size was calculated using G*Power Software (version 3.1.9.7) to ensure sufficient statistical power for detecting differences in parameter-level agreement between AI-generated and Consensus programs. Based on a previous study in a similar field and using a chi-square test for goodness of fit, the required sample size was calculated as 40 patients to achieve 80% power with a 5% significance level [16].

### Agreement Evaluation

For each rehabilitation parameter (e.g., type of exercise, modality, dosage), agreement between the AI-generated plans and the Consensus recommendations was assessed based on frequency of usage across 40 standardized patient profiles. Specifically, we calculated how often each intervention item was recommended by the Consensus group and by the AI model, expressed as a percentage of the total patient cases. Agreement was defined as a match in these usage frequencies, regardless of whether the recommendations were made for the same individual patients. Thus, the analysis reflects content-level agreement rather than case-specific alignment. This approach was selected to enable systematic comparison across a large dataset and to evaluate the general consistency of AI-generated outputs with expert-derived protocols.

### Statistical Analysis

The rehabilitation programs for each patient were analyzed across the 50 selected parameters. Each parameter was recorded in SPSS Statistics 25 as either “present” or “absent” for the Consensus program, ChatGPT-4o, and Gemini Advanced. Chi-square (χ²) tests were applied to evaluate the compatibility between AI-generated programs and the physiotherapists’ Consensus programs. Results were analyzed at a significance level of *p* < 0.05 and presented as absolute frequencies and percentages. This parameter-level analysis allowed for the identification of agreement and disagreement rates, providing a detailed evaluation of the alignment between the AI models and physiotherapist-developed programs.

## Results

Of the 52 patients referred to the study, 9 did not meet the inclusion criteria and 3 declined to participate. Therefore, 40 patients who met the criteria and provided informed consent were enrolled. (age: 53.3 ± 7.17 years, height: 166 ± 9.05 cm, weight: 67.22 ± 11.7 kg, body mass index: 24.55 ± 4.99, Kellgren-Lawrence: 24 grade 2 and 16 grade 3)

Phase 1: Cold pack and TENS (Transcutaneous electrical nerve stimulation) were recommended across all groups with high levels of agreement (Consensus: 92.5%, ChatGPT4o: 97.5%, Gemini Advanced: 95%). No significant differences were identified for these modalities. Ultrasound and its duration/frequency demonstrated notable discrepancies, being recommended significantly less by Consensus compared to both ChatGPT4o and Gemini Advanced, with both differences being statistically significant (*p* < 0.001). Notable differences were observed in the recommendations for hip mobilization exercises and their set/frequency, with Consensus suggesting them far more often compared to both ChatGPT4o and Gemini Advanced, and both differences being statistically significant (*p* < 0.001). Hip abduction exercises and hamstring curls, along with their set/frequency, showed significant differences, with Consensus recommending them far more frequently than ChatGPT4o (*p* < 0.001) and Gemini Advanced (*p* < 0.05). Criteria for transitioning from Phase 1 to Phase 2 10% increase in quadriceps and hamstring muscle strength were recommended significantly less frequently by both ChatGPT4o and Gemini Advanced compared to Consensus, with both differences being statistically significant (*p* < 0.001). The findings for Phase 1 are summarized in Table 1.

**Table 1:** Phase 1 comparison of consensus and ChatGPT4o-Gemini advanced

Variable (available %)	Consensus	ChatGPT 4o	Gemini Advanced	p1	p2	p3
Coldpack	92.5	97.5	95	0.307	0.646	0.558
Coldpack duration/frequency	92.5	90	87.5	0.694	0.366	0.496
TENS/Interferans current	92.5	97.5	95	0.307	0.646	0.558
TENS/Interferans current duration/frequency	92.5	95	85	0.679	0.355	0.542
Ultrasound	5	52.5	82.5	**< 0.001**	**< 0.001**	**0.004**
Ultrasound duration/frequency	5	47.5	77.5	**< 0.001**	**< 0.001**	**0.005**
Knee ROM exercises	97.5	92.5	95	0.307	0.558	0.646
Knee ROM exercises set/frequency	97.5	92.5	90	0.307	0.598	0.694
Quadriceps isometric exercise	95	97.5	90	0.558	0.529	0.168
Quadriceps isometric exercise set/frequency	95	90	92.5	0.398	0.646	0.694
Hip mobilization exercises	85	27.5	20	**< 0.001**	**< 0.001**	0.288
Hip mobilization exercises set/frequency	85	25	17.5	**< 0.001**	**< 0.001**	0.471
Hip abduction exercises and hamstring curl	87.5	55	65	**< 0.001**	**0.021**	0.364
Hip abduction exercises and hamstring curl set/frequency	87.5	50	62.5	**< 0.001**	**0.010**	0.262
Criteria for transition from Phase 1 to Phase 2 (No significant knee swelling)	95	92.5	90	0.679	0.398	0.694
Criteria for transition from Phase 1 to Phase 2 (Pain NRS ≤ 3)	95	97.5	92.5	0.558	0.646	0.307
Criteria for transition from Phase 1 to Phase 2 Knee ROM flexion ≥ 90 extension ≤ -5 degree.	92.5	90	97.5	0.694	0.307	0.168
Criteria for transition from Phase 1 to Phase 2 Improvement in quadriceps and hamstring muscle strength.	92.5	60	35	**< 0.001**	**< 0.001**	**0.026**

Abbreviations: p1: Consensus-ChatGPT comparison; p2: Consensus-Gemini comparison; p3: ChatGPT-Gemini comparison; TENS: Transcutaneous electrical nerve stimulation; ROM: Range of motion; NRS: Numeric rating scale

Phase 2: TENS and interferential current, including their duration and frequency, were recommended significantly more frequently by Consensus compared to both ChatGPT4o and Gemini Advanced, with the differences being statistically significant (*p* < 0.001). Balance and proprioception exercises, along with their set and frequency, were similarly recommended more frequently by both ChatGPT4o and Consensus, while Gemini Advanced’s recommendations were notably lower, with statistically significant differences observed (*p* < 0.001). Hip stabilization exercises, including their set and frequency, were recommended far more frequently by Consensus compared to both ChatGPT4o and Gemini Advanced, with all differences reaching statistical significance (*p* < 0.001). Findings for these parameters are summarized in Table 2.

**Table 2:** Phase 2 comparison of consensus and ChatGPT4o-Gemini advanced

Variable (available %)	Consensus	ChatGPT 4o	Gemini Advanced	p1	p2	p3
Hotpack	92.5	87.5	85	0.366	0.355	0.747
Hotpack duration/frequency	92.5	82.5	85	0.179	0.355	0.763
TENS/Interferans current	92.5	20	17.5	**< 0.001**	**< 0.001**	0.775
TENS/Interferans current duration/frequency	92.5	15	10	**< 0.001**	**< 0.001**	0.501
NMES/RUS current	87.5	77.5	90	0.242	0.725	0.132
NMES/RUS current duration/frequency	87.5	72.5	82.5	0.095	0.533	0.287
CKC exercises Quadriceps strengthening	97.5	95	92.5	0.558	0.307	0.646
CKC exercises Quadriceps strengthening set/frequency	97.5	92.5	87.5	0.646	0.366	0.458
Balance and proprioception exercises	90	92.5	27.5	0.694	**< 0.001**	**< 0.001**
Balance and proprioception exercises set/frequency	90	85	22.5	0.501	**< 0.001**	**< 0.001**
Lower extremity strengthening exercises	97.5	95	92.5	0.558	0.307	0.646
Lower extremity strengthening exercises set/frequency	97.5	87.5	92.5	0.091	0.773	0.458
Hip stabilization exercises	92.5	30	22.5	**< 0.001**	**< 0.001**	0.448
Hip stabilization exercises set/frequency	92.5	30	20	**< 0.001**	**< 0.001**	0.543
Hip abduction exercises and hamstring curl	70	67.5	47.5	0.844	0.092	0.137
Hip abduction exercises and hamstring curl set/frequency	70	62.5	42.5	0.562	0.041	0.144
Criteria for transition from Phase 2 to Phase 3 (No significant knee swelling)	95	92.5	80	0.646	0.071	0.158
Criteria for transition from Phase 2 to Phase 3 (Pain at a manageable level)	95	97.5	92.5	0.558	0.287	0.307
Criteria for transition from Phase 2 to Phase 3 Quadriceps and hamstring strength should support progression	92.5	90	97.5	0.694	0.773	0.168

Abbreviations: p1: Consensus-ChatGPT comparison; p2: Consensus-Gemini comparison; p3: ChatGPT-Gemini comparison; CKC: Closed Kinetic Chain; TENS: Transcutaneous electrical nerve stimulation; NMES: Neuromuscular electrical stimulation; ROM: Range of motion; NRS: Numeric rating scale

Phase 3: NMES (Neuromuscular electrical stimulation) and RUS current, including their duration and frequency, were recommended significantly more frequently by ChatGPT4o compared to both Consensus and Gemini Advanced, with all differences being statistically significant (*p* < 0.001). Dynamic balance exercises, along with their set and frequency, were recommended at similar levels by ChatGPT4o and Consensus, whereas Gemini Advanced’s recommendations were considerably lower, with statistically significant differences observed (*p* < 0.001). The findings for Phase 3 are summarized in Table 3.

**Table 3:** Phase 3 comparison of consensus and ChatGPT4o-Gemini advanced

Variable (available %)	Consensus	ChatGPT 4o	Gemini Advanced	p1	p2	p3
NMES/RUS current	17.5	65	15	**< 0.001**	0.808	**< 0.001**
NMES/RUS current duration/frequency	17.5	62.5	12.5	**< 0.001**	0.619	**< 0.001**
Quadriceps strengthening exercises	97.5	95	92.5	0.558	0.646	0.646
Quadriceps strengthening exercises set/frequency	97.5	92.5	87.5	0.646	0.122	0.366
Lower extremity strengthening exercises	90	80	85	0.286	0.572	0.639
Lower extremity strengthening set/frequency	90	77.5	80	0.132	0.286	0.823
Dynamic balance exercises	90	92.5	27.5	0.694	**< 0.001**	**< 0.001**
Dynamic balance exercises set/frequency	90	85	22.5	0.572	**< 0.001**	**< 0.001**
DLA exercises	90	87.5	97.5	0.725	0.168	0.091
DLA exercises set/frequency	90	87.5	85	0.725	0.572	0.747
Discharge criteria (pain-free functional activities)	95	92.5	97.5	0.739	0.739	0.739
Discharge criteria (muscle strength age and gender appropriate level)	95	97.5	90	0.558	0.398	0.168
Discharge criteria (significant improvement in balance and coordination ability)	95	82.5	92.5	0.078	0.646	0.179

Abbreviations: p1: Consensus-ChatGPT comparison; p2: Consensus-Gemini comparison; p3: ChatGPT-Gemini comparison; NMES: Neuromuscular electrical stimulation; DLA: Daily Life Activity

**Fig. 1:**
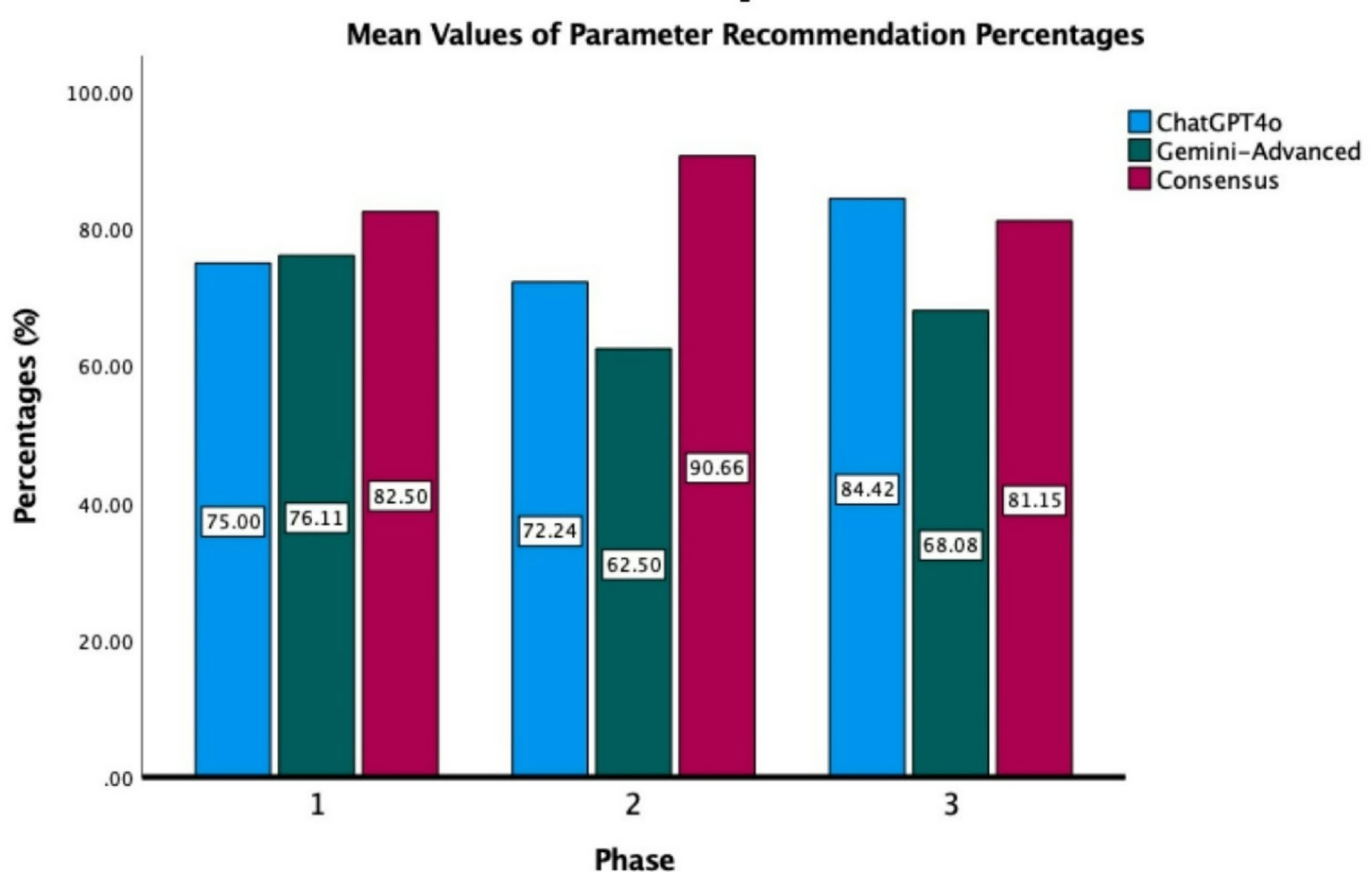
Mean values of parameter recommendation percentages

When evaluating the overall performance across the 50 different parameters in the three phases, ChatGPT4o demonstrated discrepancies with Consensus in 13 out of 50 parameters, achieving an agreement rate of 74%. In comparison, Gemini Advanced exhibited discrepancies in 15 parameters, corresponding to an agreement rate of 70%. The average percentage of recommendations for each parameter has been calculated as follows: In Phase 1, 82.5% for Consensus, 75% for ChatGPT4o, and 76.11% for Gemini Advanced; in Phase 2, 90.66% for Consensus, 72.24% for ChatGPT4o, and 62.5% for Gemini Advanced; and in Phase 3, 82.15% for Consensus, 84.42% for ChatGPT4o, and 68.08% for Gemini Advanced. The findings are illustrated in Fig. 1.

## Discussion

This study is the first to examine the potential of the large language models ChatGPT-4o and Gemini Advanced to create physiotherapy and rehabilitation programs for knee OA patients. Both models demonstrated strengths in generating general recommendations; however, they exhibited inconsistencies in terms of coverage and adherence to the Consensus. ChatGPT-4o showed greater consistency, while Gemini Advanced showed more variability in certain recommendations. These findings highlight the need for further refinement of such models to improve their reliability in clinical practice.

### Treatment Recommendation

Studies on LLMs point out their potential in general knowledge distribution, while revealing limitations in certain clinical applications. In a study examining the potential of LLMs in knee OA treatment management, language models showed limited effectiveness in tasks requiring comprehensive medical knowledge. Their performance declined when moving from general to personalized tasks. In addition, it was determined that their performance can be notably improved through the inclusion of accurate information and explicit instructions [7].

One study showed that ChatGPT-4.0 demonstrates enhanced performance in responding to general OA-related queries, exhibiting higher accuracy and compatibility in providing rehabilitation information compared to other language models [18]. Similarly, in our study, although ChatGPT-4.0 and Gemini Advanced provided significantly fewer recommendations for hip mobilization and stabilization exercises compared to the Consensus, ChatGPT-4.0 demonstrated elevated accuracy and concordance rates in designing rehabilitation programs. This difference may be due to ChatGPT4o’s and Gemini Advanced’s tendency to focus primarily on the affected area when designing rehabilitation programs and thus their clinical reasoning may not have adequately recognized the importance of hip exercises in meeting broader rehabilitation needs.

For instance, ChatGPT has been shown to effectively design rehabilitation programs based on established frameworks such as FITT-VP (Frequency, Intensity, Time, Type, Volume, and Progression) [15]. In more complex scenarios such as scoliosis classification and treatment planning, ChatGPT-4 achieved high accuracy, whereas Gemini exhibited output inconsistencies that posed potential patient safety concerns. Other studies involving various musculoskeletal conditions—such as shoulder, spine, or vestibular disorders—have reported that while LLMs can provide general information, they often lack specificity and clinical reasoning in generating detailed exercise recommendations [11–14, 29].

In our study, the language models provided significantly fewer recommendations than the consensus, particularly for hip mobilisation, stabilisation, and abduction exercises and related parameters such as sets and frequency. These findings are consistent with the literature showing the limitations of LLMs in generating specific exercise recommendations, despite their optimistic level of accuracy in designing rehabilitation programmes.

A noteworthy finding in our study was the frequent recommendation of ultrasound in Phase 1 by the language models, although it was rarely included in the Consensus recommendations. This inconsistency may be attributed to limitations in the currentness of ChatGPT’s and Gemini’s training data, as well as the models’ weaknesses in reviewing and integrating up-to-date literature [30]. Additionally, ChatGPT demonstrated strength in providing general recommendations, on the other hand it showed deficient consistency in certain exercise parameters (e.g., sets and repetitions). Similarly, while Gemini showed higher compatibility in certain clinical modalities, it struggled with defining adequate transition criteria and outcome measures. These findings underline the potential of language models as complementary tools in clinical practice and highlight the need for their optimization through the integration of more current and evidence-based datasets.

### Compatibility with Clinical Consensus

Several studies have investigated the alignment of large language models (LLMs) with evidence-based clinical guidelines for osteoarthritis, revealing noteworthy findings regarding their level of concordance.

One study compared ChatGPT and Bard (Gemini) for concordance with the American Academy of Orthopaedic Surgeons (AAOS) Clinical Practice Guidelines for hip and knee OA in terms of alignment with clinical questions. It indicated that ChatGPT achieved an 80% concordance rate with AAOS guidelines. Moreover, ChatGPT outperformed Bard, which achieved a 60% concordance rate [8].

Beyond OA, LLMs have shown varying levels of guideline adherence across other clinical areas. In this context, one study evaluated the ability of LLMs to provide treatment recommendations for rotator cuff tears and anterior cruciate ligament injuries in accordance with AAOS clinical practice guidelines, finding that ChatGPT-4 achieved a concordance rate of 79.2%, outperforming other models [31]. Similarly, studies in other specialties—such as gastrointestinal oncology and pediatric orthopedics—have reported varying degrees of guideline alignment for both ChatGPT and Gemini, generally ranging between 67% and 77% [32, 33].

In this context, the results of our study demonstrated that ChatGPT-4.0 and Gemini Advanced exhibited both strengths and weaknesses in aligning with clinical practice consensus, consistent with findings in the current literature. ChatGPT-4.0 showed deficiencies in phase 3 NMES/RUS parameters, especially in terms of duration and frequency, whereas Gemini Advanced showed lower accuracy particularly in phase 2 balance and proprioception exercises, in phase 3 dynamic balance exercises, and in set and frequency parameters. Our findings revealed that ChatGPT-4.0 achieved a 74% concordance rate with Consensus guidelines, with 13 discrepancies out of 50 parameters, while Gemini Advanced achieved a 70% concordance rate with 15 discrepancies. These results confirm ChatGPT’s relatively higher adherence to established Consensus. However, the close difference between the two models may be attributed to similar advances in the development of their premium versions. Taken together, both the literature and our findings indicate that ChatGPT-4o and Gemini Advanced demonstrate varying degrees of compatibility with clinical consensus, depending on the context. While both models perform adequately in general clinical applications, they exhibit notable limitations in specific parameters and individualized treatment planning. These findings highlight the need to enhance guideline adherence and improve the quality and currency of language model training datasets by integrating robust, evidence-based frameworks—particularly to strengthen their utility and reliability in physiotherapy and rehabilitation practice.

### Clinician Support

Current literature suggests that LLMs like ChatGPT and Gemini hold strong potential in supporting clinical practice. A 2024 study showed that ChatGPT clearly presents treatment protocols and effectively explains surgical risks [34], while another found that both ChatGPT-4 and Gemini 1.5 Pro successfully simplified ultrasound findings with high accuracy and readability [35]. Other condition-specific studies reported varying effectiveness, with ChatGPT-4 performing well for low back pain and scoliosis, whereas Gemini, though faster, showed lower accuracy in some cases [36, 37]. Our findings support existing evidence that ChatGPT-4o and Gemini Advanced, each showing over 70% accuracy, can assist clinicians in developing patient-centered rehabilitation programs for knee osteoarthritis. These models may help with assessment, treatment planning, and patient communication; however, their variable precision underscores the need for clinician oversight to ensure safe and effective use in practice.

The literature demonstrates that the most up-to-date versions of LLMs tend to exhibit superior performance as compared with lower versions [19, 38–40]. In alignment with this, we hypothesize that the high concordance observed in our study with established Consensus may be attributed to the utilization of the most up-to-date and advanced version of the model. However, discrepancies observed in certain cases may be due to limitations of the models in accessing and integrating up-to-date literature [30]. Similar to the literature, the most important limitation identified in our study may be due to the decline in performance of language models when used in languages other than English [41–43].

### Limitations and Future Work

The present study has several limitations. The best performance of language models is observed in English; however, in our study, the models were utilized in Turkish. While the treatment programs generated by AI language models raise ethical concerns, their application in clinical practice could provide clearer insights. Furthermore, our study focused solely on patients with knee osteoarthritis, limiting the generalizability of the findings. Additionally, the cross-sectional design of this study does not account for the rapid and continuous evolution of LLMs, particularly in premium versions that are regularly updated. Another limitation is that the stability of LLM-generated responses was not systematically assessed; the same input could potentially produce varying outputs at different times. Future research on the performance of language models in designing rehabilitation programs for other diseases and larger sample groups, in collaboration with physiotherapists, will allow for more comprehensive evaluations in this area. At the same time, the development of a language model specific to physiotherapy and rehabilitation may also be an extraordinary plan for the future.

## Conclusion

In conclusion, the present study contributes to the growing evidence for the potential use of ChatGPT-4o and Gemini Advanced in clinical settings, with a specific focus on the design of individualized rehabilitation programs for knee osteoarthritis. These models may support physiotherapists, physicians, and clinicians in developing personalized treatment plans. However, it is evident that there are still limitations in terms of guideline adherence and data accuracy. Further refinements are required, including improvements in language-specific performance and integration of up-to-date evidence, in order to strengthen the role of these models in the development of rehabilitation programs and broader clinical practice.

## Electronic Supplementary Material

Below is the link to the electronic supplementary material.

Supplementary Material 1

Supplementary Material 2

## Data Availability

No datasets were generated or analysed during the current study.
