# Supplementary material for: The Role of Artificial Intelligence Large Language Models in Personalized Rehabilitation Programs for Knee Osteoarthritis: An Observational Study
Source: J Med Syst. 2025 Jun 3;49(1):73. doi: 10.1007/s10916-025-02207-x (PMC12134017; doi:10.1007/s10916-025-02207-x)

**1. ChatGPT4o First Prompt (Turkish)**


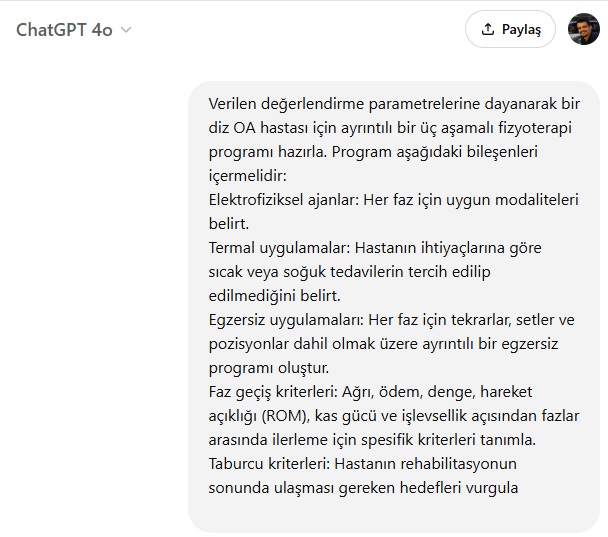


**2. Gemini Advanced First Prompt (Turkish)**


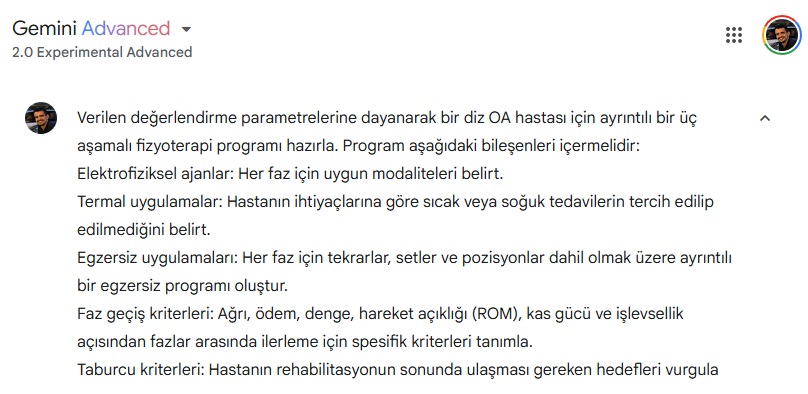


**3. ChatGPT4o patients data prompt (Turkish)**


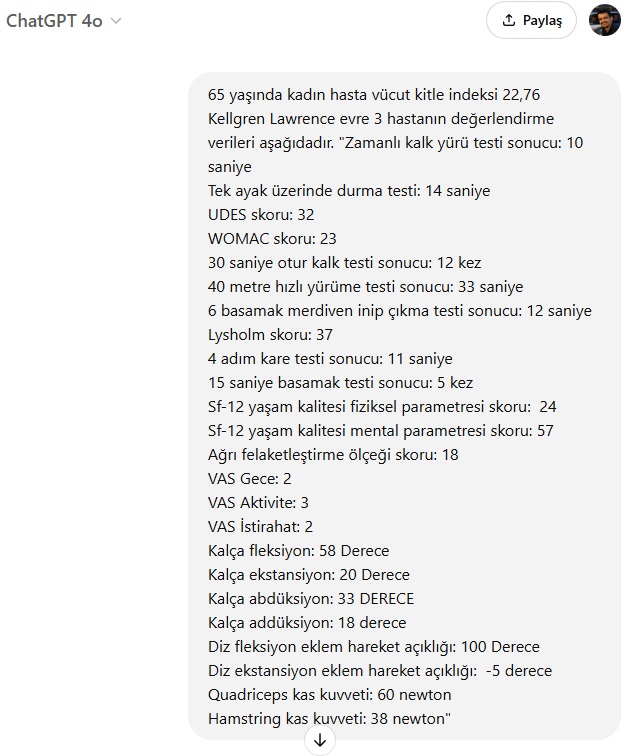


**4. ChatGPT4o patients data prompt (English)**


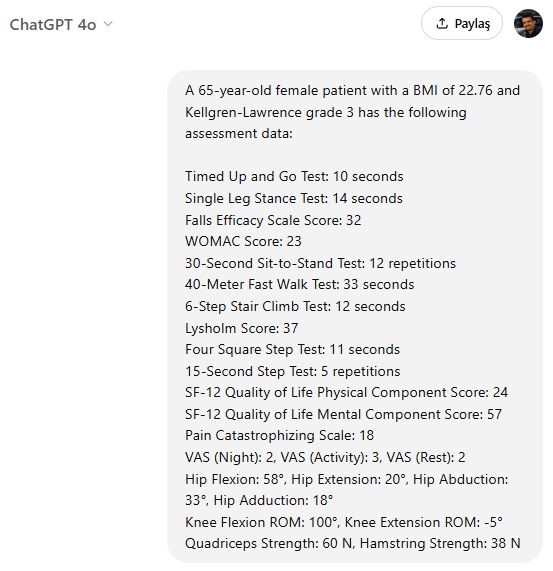


**5. Gemini Advanced patients data prompt (Turkish)**


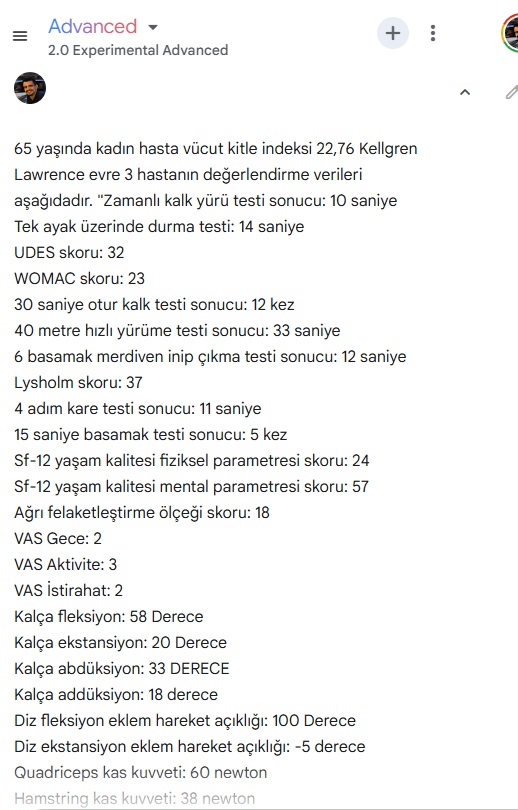


**6.** **Gemini Advanced patients data prompt (English)**


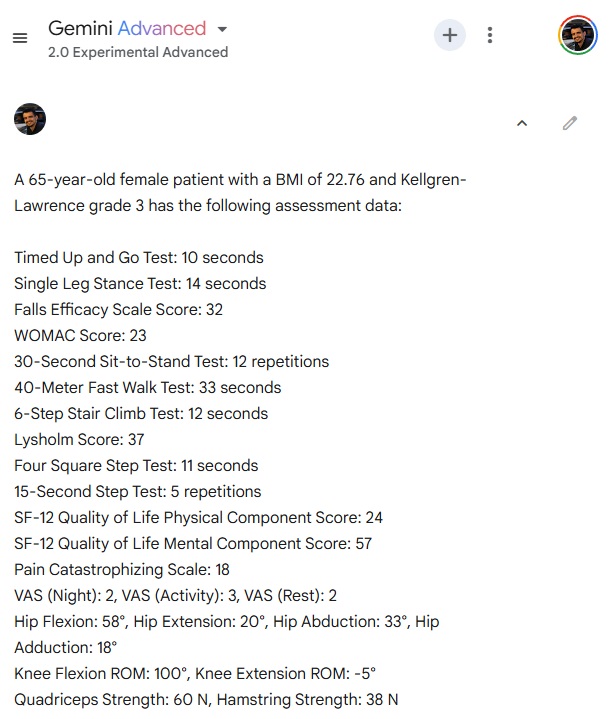

Supplement: Supplementary file 1 — Supplementary Material 1 [file 10916_2025_2207_MOESM1_ESM.docx]
