# Supplementary material for: The Role of Artificial Intelligence Large Language Models in Personalized Rehabilitation Programs for Knee Osteoarthritis: An Observational Study
Source: J Med Syst. 2025 Jun 3;49(1):73. doi: 10.1007/s10916-025-02207-x (PMC12134017; doi:10.1007/s10916-025-02207-x)
Supplement: Supplementary file 2 — Supplementary Material 2 [file 10916_2025_2207_MOESM2_ESM.docx]

**Supplement 2. Parameters excluded from those recommended by LLMs**

| **Phase** | **ChatGPT4o** | **Gemini Advanced** |
| --- | --- | --- |
| **Phase 1** | Ankle pumping exercises | Lymphatic drainage techniques |
| **Phase 1** |  | Postural training |
| **Phase 2** | Single-leg stance exercises | Orthoses |
| **Phase 2** |  | Soft tissue relaxation techniques |
| **Phase 3** | High-resistance squats | Plyometric exercises |
